# Fertility Protection, A Novel Concept: Umbilical Cord Mesenchymal Stem Cell-Derived Exosomes Protect against Chemotherapy-Induced Testicular Cytotoxicity

**DOI:** 10.3390/ijms25010060

**Published:** 2023-12-20

**Authors:** Farzana Liakath Ali, Hang-Soo Park, Analea Beckman, Adrian C. Eddy, Samar Alkhrait, Mohammad Mousaei Ghasroldasht, Ayman Al-Hendy, Omer Raheem

**Affiliations:** 1 Department of Obstetrics and Gynecology, University of Chicago, Chicago, IL 60637, USA; fliakathali@bsd.uchicago.edu (F.L.A.);; 2 Department of Surgery, University of Chicago, Chicago, IL 60637, USA

**Keywords:** mesenchymal stem cell, extracellular vesicles, exosome, fertility preservation, spermatogonial stem cell niche, testicular dysfunction, intratesticular injection

## Abstract

Currently, there is no viable option for fertility preservation in prepubertal boys. Experimentally, controlled vitrification of testicular tissue has been evaluated and found to cause potential structural damage to the spermatogonial stem cell (SSC) niche during cryopreservation. In this report, we leveraged the regenerative effect of human umbilical cord-derived Mesenchymal stem cell exosomes (h-UCMSC-Exo) to protect against testicular damage from the cytotoxic effects of polychemotherapy (CTX). A chemotherapy-induced testicular dysfunctional model was established by CTX treatment with cyclophosphamide and Busulfan in vitro (human Sertoli cells) and in prepubescent mice. We assessed the effects of the exosomes by analyzing cell proliferation assays, molecular analysis, immunohistochemistry, body weight change, serum hormone levels, and fertility rate. Our data indicates the protective effect of h-UCMSC-Exo by preserving the SSC niche and preventing testicular damage in mice. Interestingly, mice that received multiple injections of h-UCMSC-Exo showed significantly higher fertility rates and serum testosterone levels (*p* < 0.01). Our study demonstrates that h-UCMSC-Exo can potentially be a novel fertility protection approach in prepubertal boys triaged for chemotherapy treatment.

## 1. Introduction

Despite the continued advancement in treatment regimens for childhood cancers, cytotoxic cancer treatment results in a serious gonadotoxic effect. With the advent of new-generation cancer therapeutic agents, childhood cancer survival rates have increased over the past few decades, accounting for up to 88%. It is estimated that each year in the United States alone, approximately 10,000 children undergo gonadotoxic cancer therapy [1]. Children who survive after cancer therapy suffer from serious side effects of chemotherapy and radiotherapy, including infertility. In a study following 214 childhood cancer survivors for more than ten years, it was found that azoospermia was observed in 25% of patients, and oligospermia was observed in 28%. Cytotoxic chemotherapeutic agents have a deleterious effect on the cells that are mitotically active, including germ cells of the testis, disrupting the tightly regulated balance between the processes of spermiogenesis and apoptosis, resulting in gonadotoxicity [2,3]. The common adverse effects following chemotherapy include oligospermia and azoospermia, which lead to temporary or permanent male infertility [4,5]. Cyclophosphamide (CP) and Busulfan (BF) are the most common alkylating agents used in the treatment of childhood cancers, including multiple myeloma, sarcomas, chronic myeloid leukemia, and lymphomas [6,7]. Male infertility in cancer survivors not only impacts the inability to have their biological children but can also impact their emotional health, self-esteem, relationships, social image, finances, and overall well-being [8]. Therefore, pediatric cancer patients and their parents should be informed about the standard fertility preservation options before initiation of therapy.

The current clinically approved option to preserve fertility in adolescents and adults is cryopreservation of sperm. However, sperm preservation is not applicable in prepubertal boys, as spermatogenesis is not yet initiated [9]. The only fertility preservation option available to prepubertal boys is testicular tissue biopsy, followed by cryopreservation with the hope of retrieving these tissues when needed to produce mature sperm by in vitro spermatogenesis or transplantation of their cryopreserved tissues [10]. However, studies done in murine models indicated that transplantation of testicular tissue harboring spermatogonial stem cells (SSCs) has less homing efficiency. A deficiency in epigenetic reprogramming after SSC transplantation could be attributed to the damaged supportive microenvironment. Gonadotoxic chemotherapy and radiation not only damage the SSCs but also niche cells such as Leydig, Sertoli, and peritubular cells [11,12]. Other next-generation technologies are being developed for when cryopreservation of sperm and testicular tissue is not available, such as the production of induced pluripotent stem cells (iPSCs) derived from skin biopsies or blood to use for in vitro spermatogenesis [13]. Both cryopreservation and iPSC options are still in the experimental stage, and there remains a huge unmet need for preserving the SSC niche and fertility in prepubertal boys undergoing gonadotoxic chemotherapy.

Mesenchymal stem cells (MSCs) are multipotent adult stem cells isolated from various sources like bone marrow, umbilical cord, placenta, and adipose tissue. They have the ability to regenerate various damaged cells in the body, including those related to infertility [14]. The paracrine therapeutic effect of MSCs in gonadal regeneration is known to be mediated by the exosomes carrying the essential microRNAs (miRNA), cytokines, anti-inflammatory cytokines, and related proteins. These exosomes are shown to have anti-apoptotic, anti-inflammatory, and anti-oxidative properties [15,16,17]. Bone marrow-derived MSCs (BMMSCs) and Sertoli cells share the same embryonic origin, differentiation, and immunomodulatory capacity. Sertoli cells in the testes play an important role in supporting self-renewal, the maintenance of spermatogonia stemness, and spermatogenesis [18]. They play an important role in spermatogenic regulation through their gene expression and proliferation capabilities [19,20,21]. Previous studies have demonstrated the regenerative potential of MSCs in testicular dysfunction (TD) animal models. Direct injection of allogeneic BMMSCs into testes not only increased the number of spermatogonia but also repaired the testicular microenvironment in sterile rats [22,23,24,25,26]. It was also found that direct injection of BMMSCs suppresses the immune response responsible for the germ cell damage in BF-treated mice [24,27,28]. Other murine studies have also shown the gonadal regenerative ability of MSCs from bone marrow [24,25,26,29], umbilical cord [22,28,30], and adipose tissue [23,31]. MSC transplantation in the testes of chemically sterilized mice [22,28], rats [23,24,30,31], and hamsters [25,29] not only showed paracrine immune modulation [32], but also increased germ cell proliferation [22,33], angiogenesis, and the surface area of seminiferous tubules [24]. Additionally, a 42-day culture of prepubertal mouse testes showed that using the secretome, or cellular cargo, from an in vitro syngeneic BMMSC culture can be effective in promoting SSC/progenitor cell pool growth and differentiation of spermatogonia into round spermatids [34].

Exosomes, the nanosized extracellular vesicles secreted by an array of cell types, have emerged as influential entities in the domain of cell-to-cell communication. Their ability to transfer an array of bioactive molecules, including proteins, lipids, and nucleic acids, has positioned them as significant regulators in various physiological and pathological processes [35]. In the broader context of therapeutics, the inherent qualities of exosomes—such as their size and ability to interact closely with cells—enable them to be potential candidates for drug delivery [36,37]. Elliott et al. emphasized the potential of exosomes to cross biological barriers, suggesting that they could be harnessed as delivery vehicles for targeted treatments, especially in the regenerative space of male reproductive health [38]. However, previous studies used MSCs and exosomes derived from various sources to restore spermatogenesis after chemotherapy-induced testicular damage and did not address the effect of the MSCs or exosomes in protecting the SSC niche as a preventive approach before chemotherapy. In addition, none of these studies used a prepubescent rodent model to mimic childhood cancer chemotherapy. In this study, we report the protective effect of human umbilical cord-derived Mesenchymal stem cell exosomes (h-UCMSC-Exo) treatment through intra-testicular (IT) injection in a prepubescent chemotherapy-induced testicular damage mouse model. We also assessed the therapeutic potential of h-UCMSC-Exo in an in vitro cell model by comparing the effect on cell proliferation, apoptosis, and spermatogenesis marker gene expression. We also compared the effect of single versus multiple IT injections of h-UCMSC-Exos in terms of testicular function and fertility outcomes to evaluate whether the IT injections offer long-term protection of spermatogenesis.

## 2. Results

### 2.1. Effects of h-UCMSC-Exos on Human Sertoli Cells

**Figure 1:**
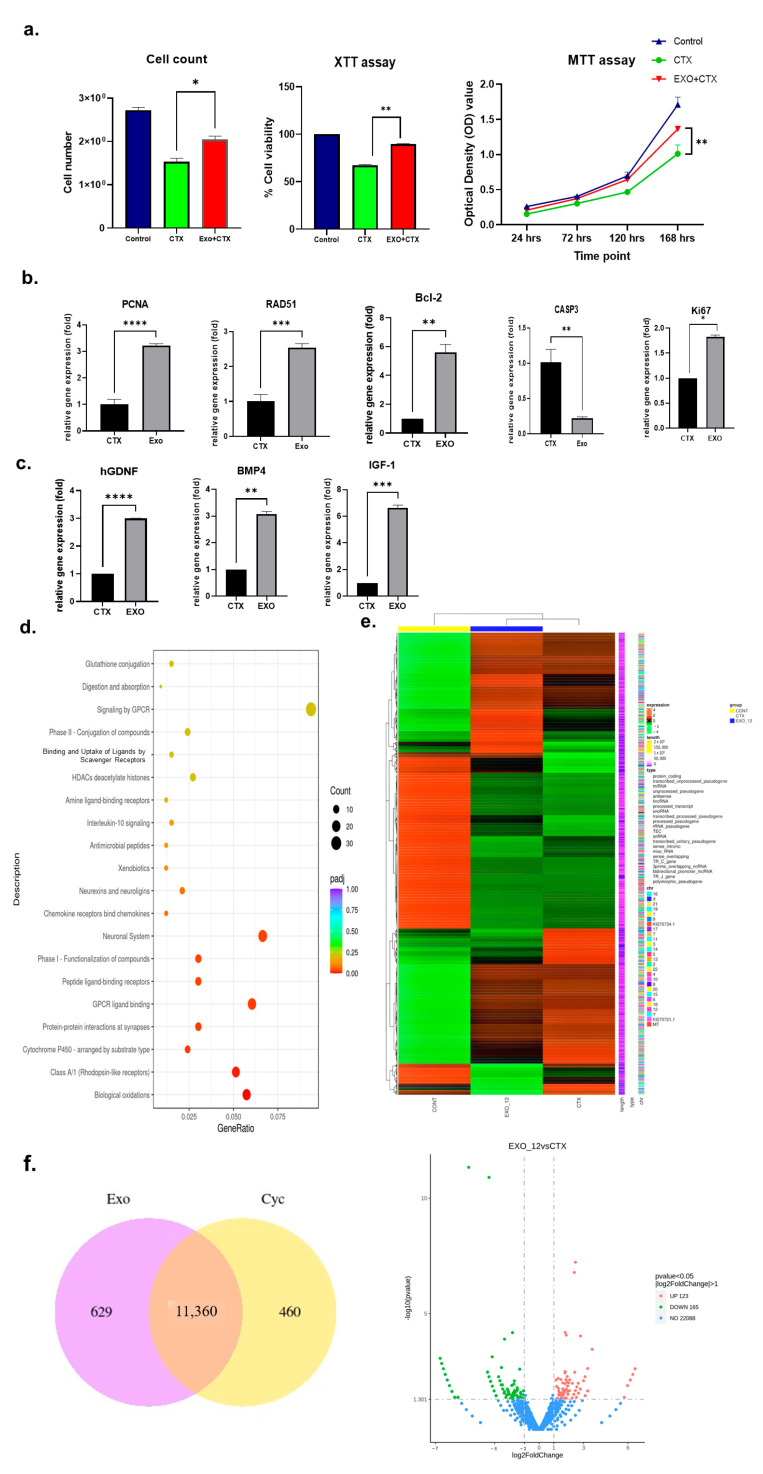
Effect of h-UCMSC-Exo on an in vitro TT model using hSerC. (**a**) Cell proliferation and viability assays. (**b**) Relative gene expression of candidate cell proliferation and apoptosis genes. (**c**) Relative gene expression of SSC markers. (**d**) Gene enrichment pathway. (**e**) Heat map of differentially expressed genes (**f**) Venn diagram of upregulated genes; Reactome pathways between CTX (polychemotherapy with CP and BF) and Exo (FP group pre-treated with h-UCMSC-Exo) groups. Data are presented as the mean ± SD (n = 3, significance level * *p* < 0.05, ** *p* < 0.005, *** *p* < 0.0005, **** *p* < 0.0001).

To evaluate the protective effects of h-UCMSC-Exo on human Sertoli cells exposed to polychemotherapy in vitro, a testicular toxicity (TT) model of human Sertoli cells (hSerC) was utilized. The fertility protection (FP) group was pre-treated with 1.5 × 10^9^ exosome particles produced from h-UCMSC-Exos for 24 h. The FP group showed improved cell proliferation and cell viability (*p* < 0.05) compared with the CTX group, as confirmed by MTT and XTT assays (Figure 1a). Among the candidate genes tested, the relative gene expression of PCNA, Ki67, Bcl-2, RAD51, and SSC markers hGDNF, BMP4, and IGF-1 in the FP group was significantly upregulated (*p* < 0.01) compared with the CTX group with a concurrent downregulation of CASP3, which indicates pro-cell proliferative, anti-apoptotic, and DNA-repair mechanisms exerted by exosomes (Figure 1b). RNA sequencing results based on the KEGG enrichment analysis confirmed the exosome uptake by endocytosis mediated by G protein-coupled receptors (GPCRs), particularly through class a/1 (rhodopsin-like receptors), and altered biological oxidation pathways in the FP group compared with CTX (Figure 1d). The differential gene expression pattern between the control, CTX, and FP groups is shown in the heatmap (Figure 1e), and differences in genes overlapping in each group or expression differences alone were confirmed through a Venn diagram (Figure 1f). Among the CTX and FP groups, 11,360 genes overlapped, 629 genes in the FP group, and 460 genes in the CTX group were differentially expressed. The volcano plots showed that among the differentially expressed genes, 123 genes were upregulated and 165 genes were downregulated (Figure 1f).

### 2.2. Pretreatment of h-UCMSC-Exo Improved Total Body Weight, Testicular Size, and Testicular Weight after Polychemotherapy-Induced TD

**Figure 2:**
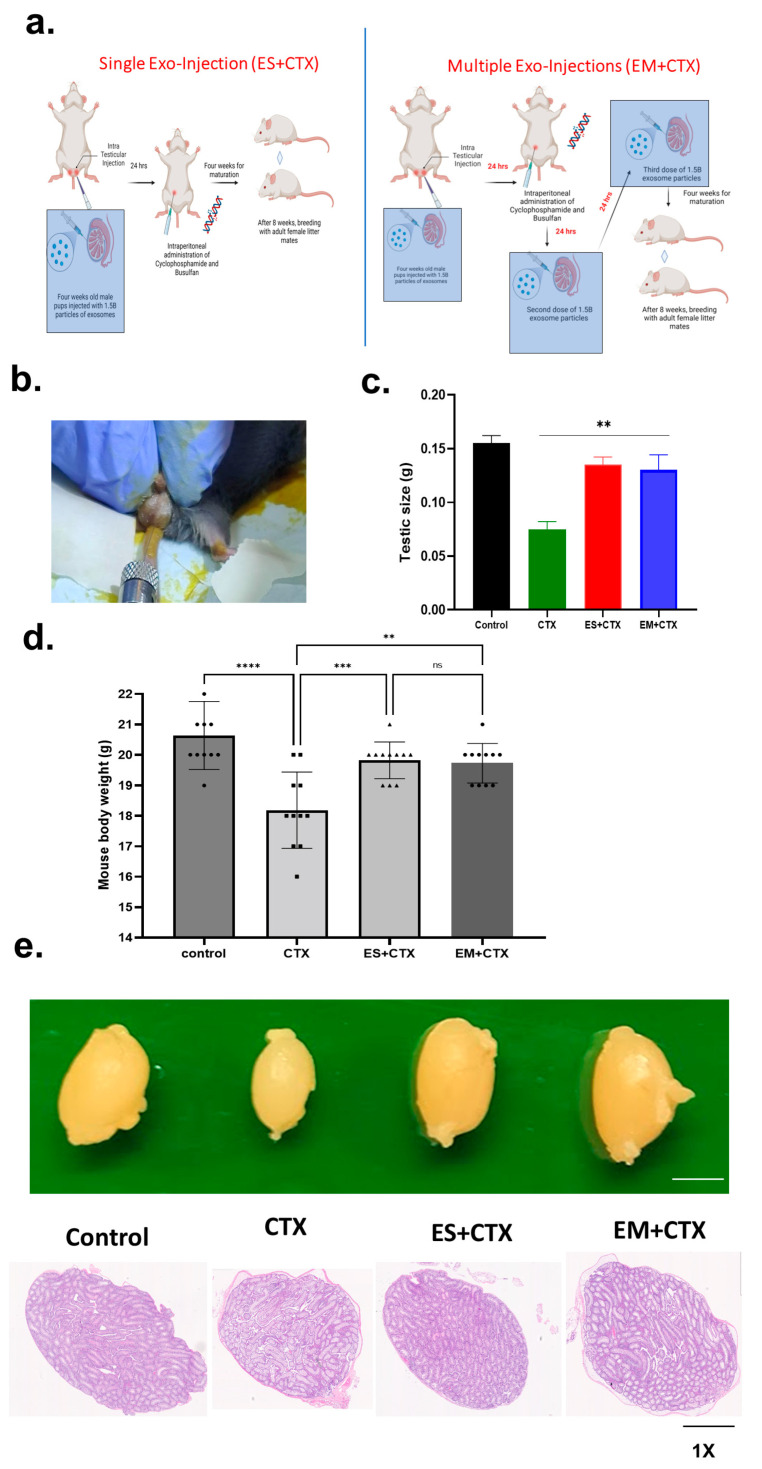
Pretreatment of h-UCMSC-Exo improved total body weight, testicular size, and testicular weight after polychemotherapy-induced testicular damage. (**a**) Single and multiple injections of h-UCMSC-Exo and CTX model. (**b**) representative image of intra-testicular injection; (**c**,**d**) changes in mouse testicular size and total body weight in all groups. (**e**) The macroscopic appearance of mouse testes in each group (scale bars: 1 mm in (**e**). Symbols on the bar graph indicate the actual body weight of each mouse. Data are presented as the mean ± SD (n = 3, significance level ** *p* < 0.005, *** *p* < 0.0005, **** *p* < 0.0001; ns: not significant).

Prepubescent male mouse colonies were successfully developed in-house by breeding healthy adult C57BL6 male and female mice. After three weeks, male pups were separated and grouped as control, CTX, Exo-single injection (ES+CTX), and exo-multiple injections (EM+CTX). At four weeks of age, both ES+CTX and EM+CTX received 1.5 × 10^9^ particles/mL of h-UCMSC-Exo in 10 µL through intra-testicular injection without an incision, 24 h before CTX intraperitoneal injection (120 mg/kg CP and 30 mg/kg BF) (Figure 1a,b). The CTX group showed significantly decreased total body weight (18.180 ± 0.7 g) compared with that of the control group mice (20.65 ± 0.15 g), ES+CTX (19.18 ± 0.54 g), and EM+CTX (19.72 ± 0.50 g) groups. To better understand whether h-UCMSC-Exo prevents TD, we compared testicular weight and size among the four groups. In comparison with the control group, the testicular weight and size were reduced by approximately 50% in the CTX group. However, the same parameters were remarkably enhanced to near-normal levels four weeks after h-UCMSC-Exo transplantation (Figure 2c,d). The macroscopic appearance of the testis in the CTX group was much smaller compared with the control and exosome pre-treated groups, and the histology of the testes showed altered morphology in the CTX group, especially loss of intersteritial cells and germinal epithelium compared with the control and exosome pre-treated groups (Figure 2e).

### 2.3. h-UCMSC-Exo Protects Testes during Polychemotherapy and Preserves Fertility in a TD Mouse Model

**Figure 3:**
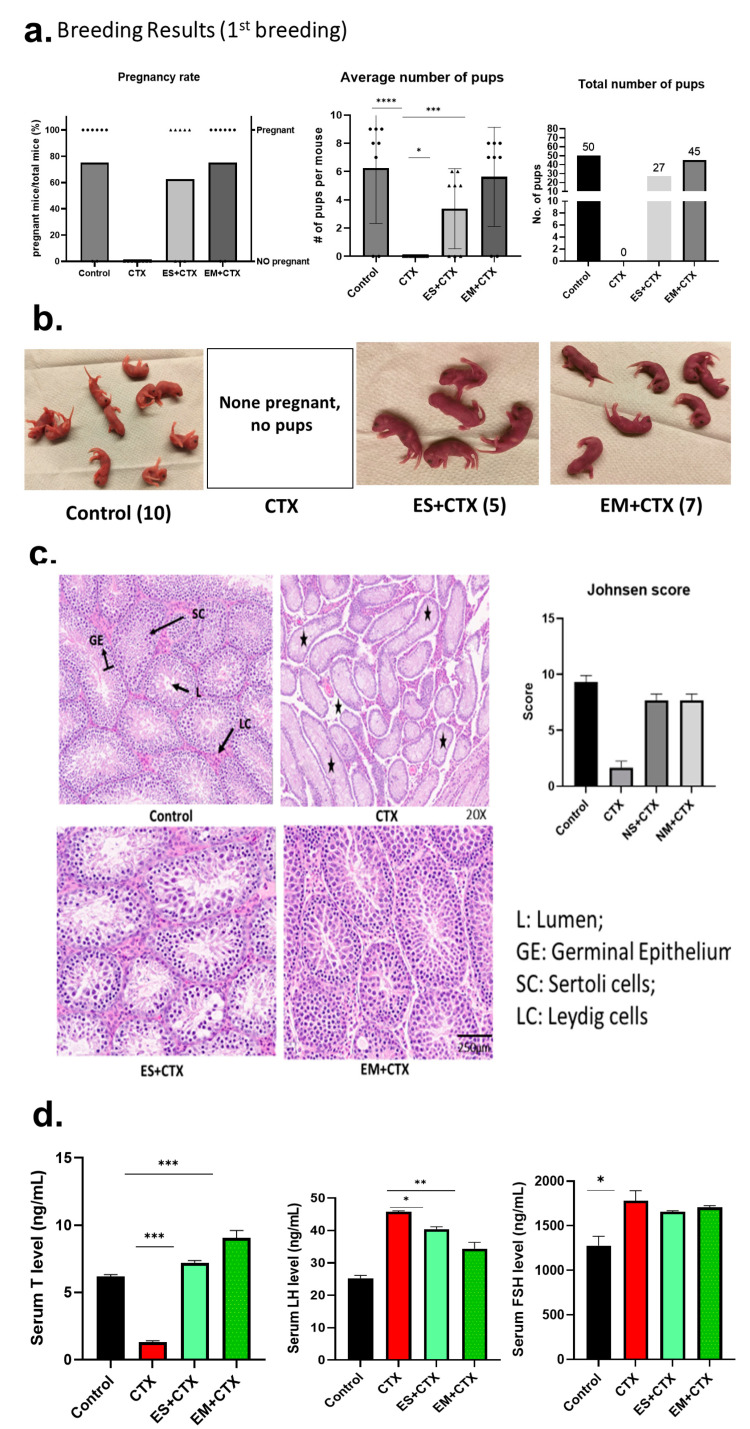
h-UCMSC-Exo protects testes during polychemotherapy and restores fertility in a polychemotherapy-induced TD mouse model. (**a**) First breeding results indicate pregnancy rates in all groups (n = 8/group). (**b**) Representative image of newborn pups on day zero in each group. (**c**) Histology of mouse testis by H&E, Johnsen’s score for testicular damage. Asterisks represent the loss of germ cells and Leydig cells in CTX group. (**d**) Serum testosterone, FSH, and LH in all experimental groups. (Scale bars: 1 mm in (**b**); 250 μm in (**c**). Symbols on the bar graph indicate the actual body weight of each mouse and # denotes the number. Asterisks represent the loss of Leydig and Sertoli cells. Data are presented as the mean ± SD (n = 3, significance level * *p* < 0.05, ** *p* < 0.005, *** *p* < 0.0005, **** *p* < 0.0001.

The breeding results indicate that no mice in the CTX group impregnated, whereas the exosome-pretreated group showed an increased pregnancy rate as well as an average number of pups. Both the healthy control and EM+CTX groups showed similar pregnancy rates (75%), followed by the ES+CTX group (62.5%) (Figure 3a). The control group delivered 9.5 ± 0.7 pups per litter, the EM+CTX group delivered 7.3 ± 0.5 pups per litter, and the ES+CTX group delivered 5.5 ± 0.25 pups per litter. Both the average and total number of pups were significantly higher in the EM+CTX group (Figure 3b) compared with the ES+CTX group. Pups were derived from the healthy control, and both exosome groups were found to be healthy and active until the day of weaning. There were no significant differences in the total body weight of pups between all groups. The testicular histology showed that the chemotherapy destroyed the germinal cells and interstitial cells in the CTX group, whereas the exosome-pretreated groups preserved testicular morphology, Leydig cells, and seminiferous tubule morphology, as indicated by the Johnsen score (Supplementary Table S1, Figure 3c). The serum hormone analysis showed chemotherapy caused permanent damage to the Leydig cells, with a significant reduction in testosterone secretion. However, the exosome-pretreated groups demonstrated decreased Leydig cell injury, with restored testosterone production (Figure 3d) at eight weeks. The other hormones, such as FSH and LH, were shown to be higher in the chemotherapy group compared with the exosome-pretreated groups.

### 2.4. h-UCMSC-Exo Preserve Ferility, Enhance Spermatogenesis-Associated Gene Markers, and Regulate Associated Pathways

**Figure 4:**
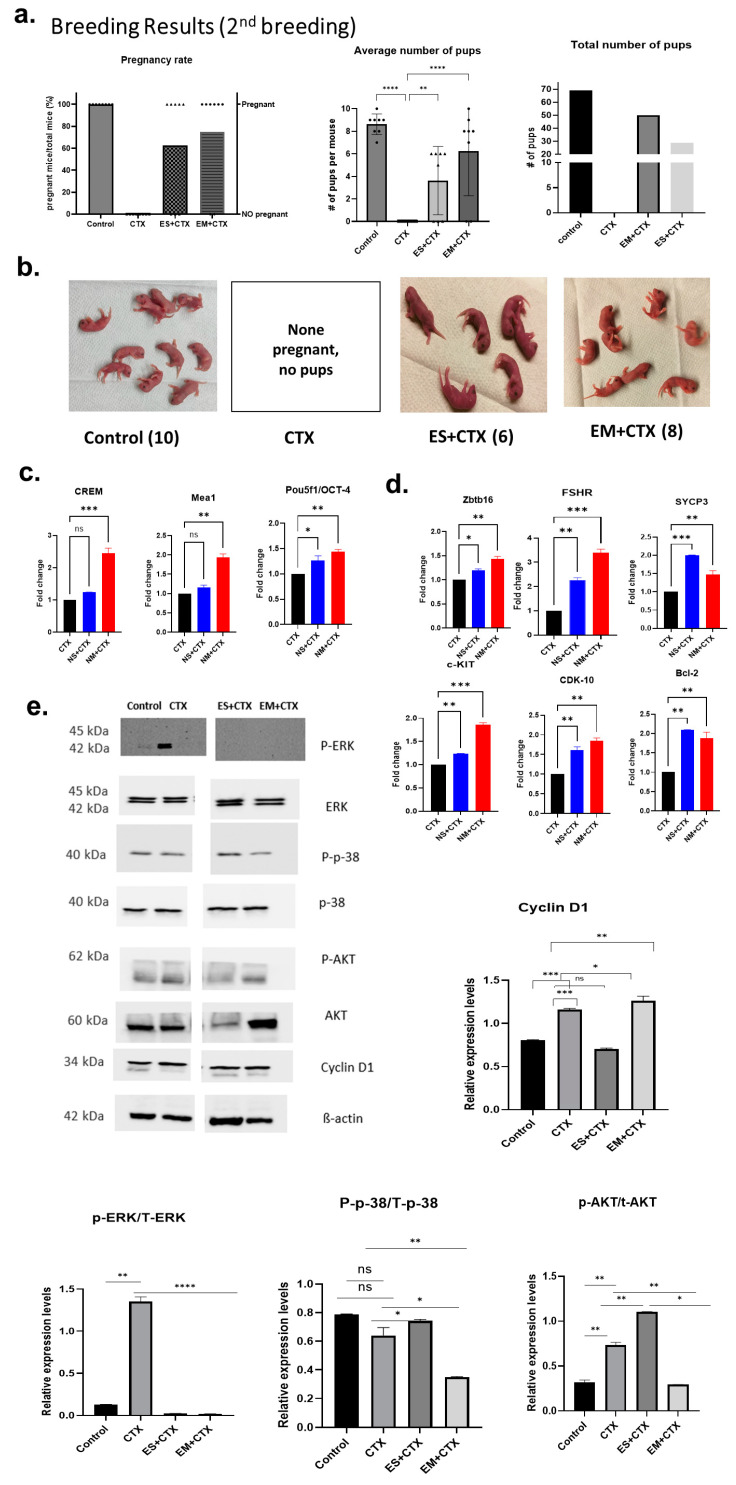
h-UCMSC-Exo restores fertility, enhances spermatogenesis-associated gene markers, and regulates associated pathways. (**a**) Second breeding results indicate pregnancy rate in all groups (n = 8/group). (**b**) Representative image of newborn pups on day 0 in each group. (**c**,**d**) Relative gene expression of spermatogenesis markers in mouse testis in each group. (**e**) Western blotting analysis of the indicated proteins in each group; quantitative analysis of phosphorylation levels of ERK, p38, and AKT in each group. C-control; CTX: CP + BF; h-UCMSC-Exo: human umbilical-cord Mesenchymal stem cell-derived exosomes; ERK: extracellular-regulated kinase; p38: p38 mitogen-activated protein kinase; AKT: protein kinase B; *p*: phosphorylation. (Scale bars: 1mm in (**b**). Symbols on the bar graph indicate the actual body weight of each mouse, and # denotes the number. Data are presented as the mean ± SD (n = 3, significance level * *p* < 0.05, ** *p* < 0.005, *** *p* < 0.0005, **** *p* < 0.0001; ns: not significant).

The second breeding experiment one month after the first breeding resulted in no pregnancy in the CTX group and similar pregnancy rates for the control (100%), EM+CTX (75%), and ES+CTX (62.5%) groups (Figure 4a). The total number of pups was 69 in the control group, 50 in the EM+CTX group, and 29 in the ES+CTX group (Figure 4a). The control group delivered 9.0 ± 0.5 pups per litter, the EM+CTX group delivered 8.0 ± 0.15 pups per litter, and the ES+CTX group delivered 6.0 ± 0.66 per litter (Figure 4b). The second breeding among the EM+CTX group suggests that multiple exosome IT injections combine to protect the testes like a molecular shield, counteracting the testciular toxicity before and after chemotherapy. To explore the potential degree of spermatogenic function recovery by h-UCMSC-Exo, the germ cell-specific (GCS) candidate genes that play a key role in spermatogenesis were analyzed for mRNA expression in the mouse testiuclar tissues. Among the genes tested, CDK-10, CREM, Mea1, Pou5f1, and ZBTB16 were significantly upregulated (*p* < 0.001) in the EM+CTX group compared with the ES+CTX and CTX groups (Figure 4c). However, SYCP3, which is expressed intensely in the nuclei of mature spermatocytes, and Bcl-2, an anti-apoptotic marker, were significantly upregulated only in the ES+CTX group (Figure 4d). The central marker genes of spermatogensis, such as FSHR and C-Kit, were found to be more expressed in the EM+CTX group (Figure 4d). The breeding results, histology, and gene expression data indicate that single and multiple injections of exosomes as pretreatment have a protective effect against CTX-induced gonadotoxicity. To evaluate the possible molecular mechanisms underlying the protective effect, we assessed the phosphorylation levels of several signaling pathway proteins. Western blot results showed that the exosomes could potentially inhibit the p38 MAPK/ERK signaling pathway, which could improve germ cell chemotherapy tolerance (Figure 4e). The phosphorylation levels of p38 MAPK, AKT, and ERK proteins were significantly elevated in the CTX group (*p* < 0.001), whereas the opposite was observed with the cell proliferation marker Cyclin D1. In contrast, both single and multiple h-UCMSC-Exo pretreatment groups showed significant reductions in the phosphorylation of p38, AKT, and ERK proteins (*p* < 0.01). The EM+CTX group showed the most significant reduction in phosphorylation levels for all three pathways (Figure 4e). These results suggest that exosomes could alleviate the reproductive toxicity of CTX, most likely through the p38 MAPK/ERK/AKT signaling pathways.

### 2.5. h-UCMSC-Exo Permanently Restore Ferility through Continued Breeding and Alleviate Testicular Damage via Various Biological Pathways

**Figure 5:**
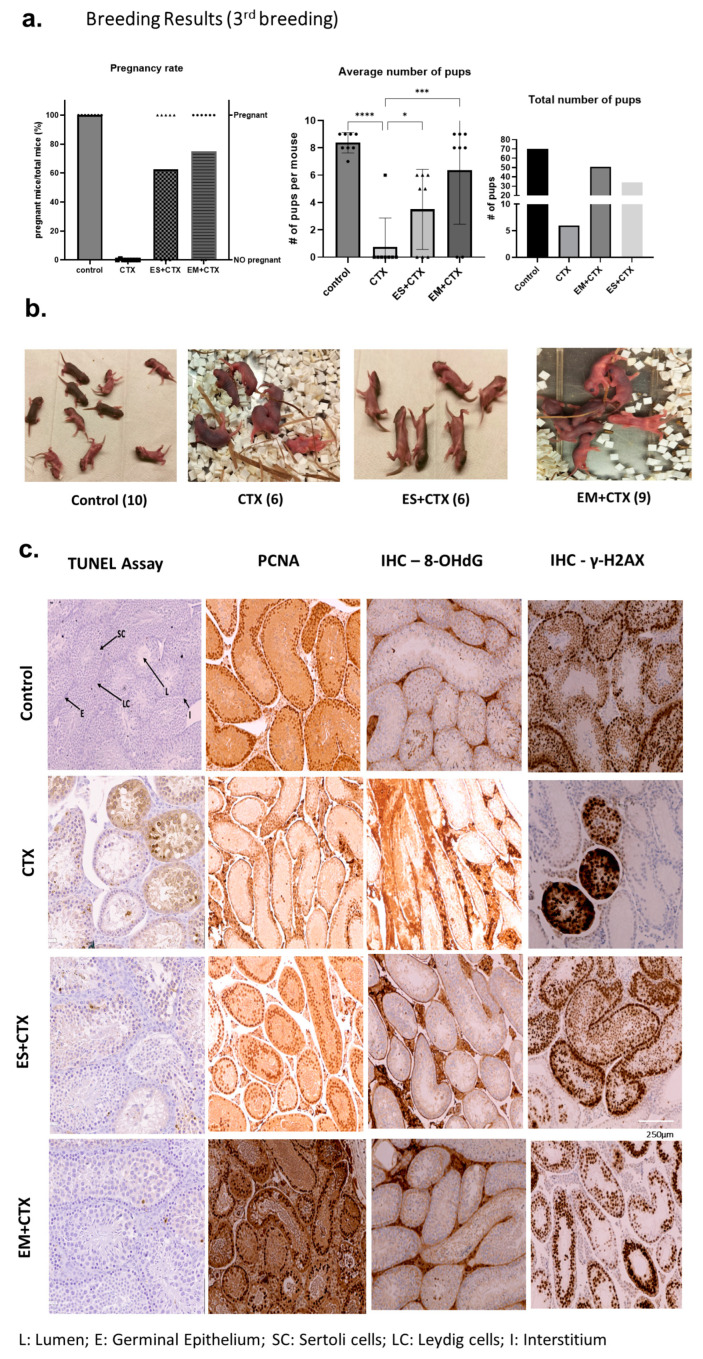
h-UCMSC-Exo permanently restores fertility throughout continued breeding and alleviates testicular damage via various biological pathways. (**a**) Third breeding results indicate pregnancy rates in all groups (n = 8/group). (**b**) Representative image of newborn pups on day 1 in each group. (**c**) TUNEL assay, IHC of PCNA, 8-OHdG, and γ-H2AX in all the groups. C-control; CTX: CP + BF; h-UCMSC-Exo: human Umbilical-Cord Mesenchymal Stem cell-derived exosomes; PCNA—proliferating cell nuclear antigen; 8-OHdG—8-Hydroxyguanosine; γ-H2AX—gamma H2A histone family member X. (Scale bars: 1mm in (**b**); 250 μm in (**c**)). Symbols on the bar graph indicate the actual body weight of each mouse and # denotes the number. Data are presented as the mean ± SD (n = 3, significance level * *p* < 0.05, *** *p* < 0.0005, **** *p* < 0.0001.

The third breeding experiment resulted in one out of eight mice becoming pregnant among the CTX group, but the other groups had similar pregnancy rates as the first and second breeding cycles, i.e., control (100%), EM+CTX (75%), and ES+CTX (62.5%). (Figure 5a). The control group delivered 9.0 ± 0.23 pups per litter, the EM+CTX group delivered 9.0 ± 0.90 pups per litter, the ES+CTX group delivered 6.0 ± 0.5 pups per litter, and the CTX group delivered 6.00 pups per litter (Figure 5b). The TUNEL assay and IHC were performed to detect anti-apoptotic and other biological pathways responsible for the protective effect of h-UCMSC-Exo. EM+CTX and ES+CTX groups showed less apoptosis in both germinal epithelium and interstitial Leydig cells, whereas the apoptotic index was higher in the CTX group (Figure 5c), quantified as shown with the apoptotic score index (Supplementary Figure S2). For the control and exosome-pretreated groups, PCNA was detected in the mitotically proliferating spermatogonia but not in spermatocytes that had just entered meiosis (Figure 5c). The testicular damage was evident in the CTX group by the 8-hydroxy-2′-deoxyguanosine, which is an important marker for measuring endogenous oxidative damage to DNA. Both EM+CTX as well as ES+CTX showed minimal DNA oxidative damage, close to the control group levels (Figure 5c). IHC of gamma H2A histone family member X (γ-H2AX), a key marker of DNA double strand breaks and genomic instability, was expressed intensely in the CTX group compared with both exosome-pretreated groups, suggesting the potential role of h-UCMSC-Exo in mediating DNA repair (Figure 5c).

## 3. Discussion

Studies on BF-induced testis toxicity in mice indicated that conditioned media from BMMSCs and exosomes from urine-derived stem cells restored spermatogenesis by facilitating intercellular adhesion molecules, reducing apoptosis, and upregulating key genes associated with spermatogenesis [39,40]. Another study on rats demonstrated that tail vein injection of BMMSC-exosomes counteracted the reproductive toxicity of CP-induced testicular damage and reversed the pathological changes through the p38MAPK/ERK and AKT signaling pathways, indicating the potential therapeutic efficiency of BMMSC-exosomes [41]. A recent study showed that exosomes derived from BMMSCs ameliorated CP-induced testosterone deficiency by enhancing the autophagy of Leydig cells through activating the AMPK-mTOR signaling pathway [42]. Intra-testicular administration of amniotic fluid-exosomes (AF-Exos) in a non-obstructive azoospermia rat model showed that both 10 and 40 μg of AF-Exos restored spermatogenesis, improved the sperm motility, microstructure of the seminiferous tubules, and expression of SSC markers in the exosome-treated group [43]. With recent advancements in cancer therapy, most cancers in children and young adults are treatable, and long-term survival is achievable. However, most pre- and post-pubertal patients receiving gonadotoxic therapy suffer from infertility [44,45], and more than 50% of children receiving chemotherapy such as CP have been reported to develop azoospermia and oligospermia in their adulthood [46]. Testicular cryopreservation is currently the only available fertility preservation option for prepubertal boys, but here we leveraged the regenerative potential of h-UCMSC-Exo to protect gonadal toxicity during chemotherapy and restore the SSC niche, which is essential for spermatogenesis in adulthood. To evaluate the effect of h-UCMSC-Exo on fertility protection, we used both in vitro TT and in vivo TD models. Our in vitro TT model used human Sertoli cells exposed to CTX (CP + BF) for 24 h, and our in vivo model used an in-house prepubescent male pup colony to recapitulate childhood cancer therapy.

During spermatogenesis, cell proliferation and apoptosis ratios are tightly regulated to maintain homeostasis. Any imbalance within the Sertoli niche results in male fertility dysfunction [47]. A previous study using an organotypic culture system of human fetal and prepubertal testis demonstrated that cisplatin exposure resulted in loss of total germ cell number, including SSC, confirming its direct relevance in children undergoing chemotherapy [48]. Our in vitro TT model showed that pretreatment with h-UCMSC-Exo enhanced cell proliferation and prevented apoptosis. Similar results were reported: MSCs derived from human amnion enhanced cell proliferation, reduced apoptosis and oxidative damage, and augmented oxidative defense against BF-induced testis toxicity in mouse Sertoli cells [49]. Another study indicated that co-culturing adipose-derived MSCs with Sertoli cells under retinoic acid and testosterone induction could stimulate the proliferation of male germ-like cells with an upregulation of germ cell-related markers such as Oct4, Stella, Ddx4, Dazl, PGP9.5, Stra8, and ITGα6. This suggests the potential therapeutic role of MSCs in male infertility-related disorders [50]. Growth factors such as BMP-4, GDNF, and IGF-1, which are essential for self-renewal and maintenance of SSC [51], were shown to be upregulated in the FP group in the present study. Our RNA sequencing results suggest altered MAPK and RAS signaling pathways in the FP group; this corroborates a well-known signal transduction mechanism of these two pathways in response to extracellular milieu cellular signals responsible for cell growth, division, and differentiation. A recent in vitro study on mouse SSCs showed that the addition of Sertoli cell-derived exosomes containing miR-30a-5p enhanced SSC proliferation and differentiation by activating the mitogen-activated protein kinase (MAPK) signaling pathway [52]. In humans, chemotherapy and/or radiotherapy result in the depletion of SSCs as well as the altered microenvironment of niche cells [53]. Chemotherapy-induced muscle wasting in cachectic mice has been well established in non-tumor-bearing rodents through various biological mechanisms, including mitochondrial depletion and activation of ERK1/2 and MAPK signaling pathways [54,55]. For our in vivo TD model, we used four-week-old male prepubescent mice to recaptulate childhood cancer patients receiving chemothrapy. h-UCMSC-Exo were injected via the intra-testcular route before CTX (intraperitoneal injection of 120 mg/kg CP and 30 mg/kg BF). Our results suggest that reductions in the total body weight as well as the testis weight caused by CTX were preserved significantly by single and multiple doses of exosome pretreatment. Goosens et al. used a similar mouse model and observed similar long-term results; intraperitoneal injection of 40 mg/kg BF followed by CdCl2 (2 mg/kg) at 8 weeks of age was established to ensure no endogenous spermatogenesis recovery. Co-transplantation of MSCs with SSCs significantly increased testis size and the testis-to-body weight ratio in transplanted groups compared with mice that received only chemotherapy [56]. In the present study, testis histology confirmed the altered tubular and entire germinal epitheilum structure in the CTX group, whereas both single and multiple injections of exosomes preserved the testicular morphology. Similar results have been reported with the transplantation of Sertoli and MSCs from syngeneic mice, which aligned as neo-tublues, leading to restored microarchitecture in a TD mouse model [57].

A fertility protection study in male prepubertal rats used MSCs to protect against Doxorubicin (DOX)-induced TT. A single intravenous administration of two million MSCs counteracted the deleterious effects of DOX on body and testicular weight, sperm quality, and fertility outcomes, similar to our study [58]. Testicular transplantation of 200 million hypoxic BM-MSCs improved fertility as well as restored the structure of primary and secondary spermatocytes, spermatid, Sertoli, and Leydig cells in an adult infertile rat model [59]. A recent study in rats showed that CP caused testicular damage, resulting in reduced serum testosterone concentration and thus increased pituitary gland production of the hormones FSH and LH. The intra-testicular administration of BM-MSC reversed the hormonal levels to those of healthy controls [60]. Our serum hormone analysis shows similar results, except for FSH levels, which should be studied further. Another study of an X-irradiation-induced testicular injury demonstrated that intra-testicular injection of hA-MSCs and conditioned media restored serum testosterone levels, increased the number of offspring, increased seminiferous tubule diameter, and increased seminiferous epithelial height. It also decreased serum FSH and LH levels, confirming the role of MSCs in aiding spermatogenesis [61]. Other reports also confirmed the therapeutic potential of BM-MSCs and human placental extracts to restore spermatogenesis and serum hormonal levels in DOX-induced and endocrine disruptor-induced TT rat models [62,63]. These reports highlight the regenerative abilities of MSCs (i.e., anti-apoptotic, anti-inflammatory, anti-oxidative stress, DNA repair enhancement) that might contribute to their ferility restoration abilities. However, authors also believe that MSCs could potentially differentiate into germ cells and sperm based on the in vivo testicular microenvironment and hence restore testicular functions in that way [64]. However, the present study clearly suggests the absence of this differentiation phenomenon altogether. Rather, we show that exosomes are responsible for preventing testicular injury during chemotherapy and thus preserving ferility.

Exosomes derived from stem cells, such as MSCs, can circulate across blood-brain or blood-testis barriers and can possess stable cellular cargo with unique intrinsic molecues based on their origin. They can be targeted towards specific cells or organs when tagged, which makes them ideal as a next-generation cell-free drug carrier. Intra-testicular injection of 10 IU of blood-serum-derived exosomes alleviated acute inflammation by reducing levels of nitric oxide (NO), malondialdehyde (MDA), and apoptotic gene expression and restoring testosterone levels, leading to the production of healthy sperm [65]. Upregulation of several germ cell markers involved in SSC self-renewal, such as OCT-4, CREM, ZBTB16, and SYCP3, in mouse testicular tissue suggests the role of injected exosomes in mediating long-term protection of SSC during cytotoxic chemotherapy exposure. A previous study showed that human embryonic stem cell-derived exosomes alleviated retinal degeneration by upregulating Oct4 in Müller cells [66].

The AKT pathway plays a critical role in cell survival, regulation of apoptosis, and metabolism [67]. It was also found to be associated with CP-induced toxicity of the heart and ovary [68,69]. ERK is generally involved in the regulation of cell growth and differentiation, but p38 MAPK is well known to be activated by stimulating factors, including chemotherapeutic agents [70]. More importantly, the ERK pathway is activated during disruption of the blood–testis barrier, resulting in spermatogenic dysfunction by regulating downstream signal molecules like the tight junction proteins occludin, connexin 43, and N-cadherin [71]. It was reported that inhibition of the p38 MAPK/ERK signal pathway reduced apoptosis and CP-induced renal oxidative stress while promoting CP tolerance by germ cells as well as its metabolite acrolein [72,73]. It was observed that BM-MSC-derived exosomes inhibited p38 MAPK/ERK and AKT signaling pathways against the reproductive toxicity of CP in a rat model [41]. The CTX group in our present study also showed elevated phosphorylation levels of p38 MAPK/ERK and AKT, but multiple exosome injections reduced the phosphorylation significantly compared with a single injection, which is related to fertility outcome and the number of pups. Pregnancy and delivery of healthy pups only occurred in the CTX group during the third breeding cycle, which strongly suggests endogenous recovery of SSC and niche through other innate regenerative mechanisms six months after CTX exposure without any exosome pre-treatment or treatment.

Though this report might be the first to harness the protective effect of exosomes on male fertility, the authors acknowledge a few limitations of the present study. We did not use a negative control such as conditioned media produced from h-UCMSCs deviating from exosomes. The addition of GW4869 to the h-UCMSCs would have inhibited the production of exosomes, and it would have confirmed the protective effect of exosomes as a primary source for testicular regeneration. Another limitation of this study is that we did not track the injected h-UCMSC-Exo with fluorescent-tagged dyes to monitor the fate of the injected exosomes in the testicular space. The bioluminescence and PET-CT scans with time-lapse imaging would have provided important data on real-time monitoring of exosomes dispersing into the subcutaneous region of the testis, uptake by the testicular niche, and shelf life of injected exosomes for potential safety-related toxicity studies in the future.

## 4. Materials and Methods

### 4.1. In Vitro Testicular Toxicity (TT) Cell Model Using Human Sertoli Cells (hSerC)

Human SerC was used as an in vitro cell TT model to test the protective effect of h-UCMSC-Exo. These are primary cells isolated from human testis and characterized by the presence of specific hSerC markers such as GATA-4 and Sox-9. They are generally used as an in vitro model to understand testicular dysgenesis and to develop treatments for male reproductive disorders. We purchased these cells from ScienCell (Carlsbad, CA, 92008, USA, cat. no. 4520) Research Laboratories and cultured them with the recommended protocol. Briefly, T75 cell culture flasks were precoated with a poly-L-lysine-coated culture vessel (2 μg/cm^2^) (Millipore, MA, USA, cat.no P4707), and hSerC were cultured using hSerC complete medium (SerCM, Cat. #4521, Sciencell Research Laboratories, LA, USA) (approximately 5000 cells/cm^2^) in a 37 °C, 5% CO_2_ incubator. The healthy hSerC were used as controls, and a TT model of hSerC was established by inducing apoptosis using 250 µg/mL of CP and 30 µg/mL BF based on the previous published literature from our lab [74]. Before inducing CP-BF-induced damage, 1.50 × 10^9^ h-UCMSC exosomes were added as a fertility protection approach to the hSerC for 24 h. The effects of exosomes on cell proliferation, DNA damage repair, and apoptosis were assessed at the end of 24 h.

### 4.2. Preparation of the h-UCMSC−Derived Exosomes

h-UCMSCs were purchased from Roosterbio (Frederick, MD, USA) and were cultured as per the manufacturer’s instructions. At passage 3, after reaching 80% confluency, cells were then washed three times with phosphate-buffered saline (PBS) to completely remove serum. A recommended collection medium for EVs, RoosterCollect™-EV (xeno-free, protein-free, and chemically defined), was added, and cells were incubated for 48 h. The collected conditioned media was centrifuged for 5 min with 500 G at 4 °C to remove the cell debris, followed by 2000× *g* for 20 min to remove apoptotic bodies, and then at 10,000× g for 30 min to remove microvesicles. h-UCMSC-derived exosomes were isolated from the supernatant using the Poly-Ethyl Glycol (PEG)-based precipitation method using a commercialized reagent (ExoQuick-TC, System Biosciences, Palo Alto, CA, USA). Isolated exosomes were stored at −80 °C for use in further experiments. The quality and quantity were analyzed using Nano Tracking Particle Analysis (Supplementary Figure S1) by NanoSight NS300 (Malvern analytical, Great Malvern, UK).

### 4.3. MTT and XTT Assays

hSerC were plated in a 96-well flat cell culture plate (CLS3799-50EA, Corning, NY, USA) at a density of 5 × 10^4^ per well for 24 h at 37 °C in a 5% CO_2_ incubator. Exosomes (100 µg/mL) isolated from h-UCMSC were added to treat CP-BF-induced damaged hSerC for 24 h. Supernatants from each well were discarded, followed by the addition of a 5 mg/mL 3-(4,5-Dimethyl-2-thiazolyl)-2,5-diphenyl-2H-tetrazolium bromide (MTT, methylthiazolyldiphenyl-tetrazolium bromide) solution. After incubation for 4 h, 200 µL of dimethyl sulfoxide (Merck, Darmstadt, Germany) was added. Next, the plates were gently shaken for 15 min at room temperature. The absorbance of each sample was measured at 570 nm using a SpectraMax Versa microplate reader (Molecular Devices, San Jose, CA, USA).

hSerC were seeded in complete medium on 96-well plates, and after 24 h, the medium was changed to serum-free SerC medium with 1% non-essential amino acids (negative control), hSerC medium with 1% non-essential amino acids and 10% serum (positive control), and hSerC medium with 1% non-essential amino acids and exosomes (100 µg/mL) isolated from h-UCMSC. The cells were incubated for 3 days in the presence of two increasing concentrations of Exo: 1× (donor-to-acceptor cell ratio 7:1) and 2× (donor-to-acceptor cell ratio 14:1). Cell proliferation was evaluated using the XTT assay viability/proliferation kit (Thermo Scientific, Waltham, MA, USA), according to the manufacturer’s instructions, and the results were reported as a percentage of the positive control.

### 4.4. RNA Extraction and Quantitative Real-Time PCR

Total RNA was extracted from hSerC and mouse testis using the Qiagen RNeasy Micro Kit (Cat. No. 74004), and cDNA was synthesized starting from 1 μg of total RNA employing the PrimeScript 1st strand cDNA Synthesis Kit (Takara Bio, San Jose, CA, USA). Real-time PCR was performed using iTaq™ Universal SYBR^®^ Green Supermix (Cat. no 1725124, Bio-Rad, CA, USA) with optimized amplification conditions on the Bio-Rad CFX96 Touch Real-Time PCR qPCR system—GL SM100063 (Bio-Rad, CA, USA). The experiments were performed in triplicate for each gene. The primers that are used for hSerC and mouse testicular tissue sequences are given in Supplementary Table S2. The analysis was performed using the comparative CT method, and GAPDH was employed for internal normalization.

### 4.5. Western Blot

Mouse testis samples were lysed using RIPA buffer (Sigma Aldrich, St. Louis, MO, USA) containing a protease inhibitor cocktail (Sigma Aldrich, St. Louis, MO, USA), and 10 µg/mL total protein was separated on 10% polyacrylamide gel via SDS-PAGE, followed by transfer on methanol-activated PVDF membrane (Merck Millipore, Burlington, MA, USA). The membranes were blocked in 5% non-fat powdered milk for 1 h at room temperature, after which they were incubated with primary antibodies to Akt (pS473) + total Akt Kit (ab126433, abcam, Cambridge, UK), ERK1 (phosphor) + total ERK (ab126445, abcam), p38 MAPK alpha + total p38 MAPK alpha (ab126453, abcam), cyclinD1 (ab16663, abcam) and β-actin (A5441, Sigma Aldrich, St. Louis, MO, USA), overnight, at 4 °C. The next day, after 3 washes with 0.1% PBST, the membranes were incubated with the corresponding secondary antibodies: goat α-mouse HRP or goat α-rabbit HRP (Thermo Scientific) for 1 h at room temperature. The membranes were developed with Immobilon Forte HRP Substrate (Merck Millipore, Burlington, MA, USA) using a Luminescent Image analyzer LAS-3000 (FUJIFILM, Tokyo, Japan). The relative protein expression was quantified via densitometric analysis with the Image J software (Version 1.53r).

### 4.6. RNA Sequencing

RNA extraction from control, CTX, and h-UCMSC-Exo-treated hSerC cells was performed, and quality control was checked for each sample. The RNA-seq assay was performed by Novogene Company (San Diego, CA, USA) as per the established protocol (Novogene mRNA-seq Services). Briefly, the appropriate library was prepared according to human SerC, followed by 150 bp paired-end sequencing. The sequencing data were stored for further bioinformatic analyses. All data from high-throughput sequencing platforms (Illumina, San Diego, CA) were transformed into sequenced reads by CASAVA base recognition (base calling). All analyses, including gene expression qualification, differential expression analysis, and functional analyses (enrichment analysis, oncogene analysis, and protein–protein interaction analysis), were performed by the bioinformatics team at Novogene.

### 4.7. Development of In-House Prepubescent Mice Colony and Mouse TD Model

The animal experiments were approved by the University of Chicago Animal Care Committee (UC IACUC). The animal protocol entitled “Study for treat female reproductive disease using small animal model” was approved by UC IACUC on 20 June 2023 (approval number 72638-25). All the animal experiments were performed in compliance with the University of Chicago’s policies and guidelines for the use of laboratory animals. Eight-week-old male and female C57BL/6 mice were purchased from Charles River (MA, USA) for the animal experiments. Mice were housed in an animal facility for at least 72 h under specific pathogen-free conditions. Healthy pups were obtained by crossing male inbred C57BL/6 with female inbred, and pre-pubertal male pups were separated after a day of weaning.

At four weeks of age, mice were randomly grouped as (1) healthy control (healthy mice with no experimental manipulations), (2) CTX (mice injected with CF+BF), (3) ES+CTX (one dose of intra-testicular injection of 1.5 × 10^9^ of h-UCMSC-Exo 24 h before CTX), and (4) EM+CTX (one dose of intra-testicular injection of 1.5 × 10^9^ of h-UCMSC-Exosomes 24 h before CTX, the second dose 24 h after CTX, and the third dose 24 h after second dose). Each group had 6 mice for breeding and four for experimental read-outs. The CTX group mimicking children receiving chemotherapy and TD in vivo model was created by injecting CF (120mg/kg bw) with BF (30 mg/kg bw) through intraperitoneal injection based on our previous results inducing Premature Ovarian Insufficiency (POI) model in female mice [74]. The FP exosome groups (both single and multiple) were preoperatively treated with a single dose of meloxicam (5 mg/kg) and were kept under anesthesia with 1–4% inhalation of isoflurane during the intra-testicular injection procedure. Ten µL of h-UCMSC-Exo containing 1.5 × 10^9^ exosome particles were injected into the scrotum. In this procedure, the skin of scrotum was cleaned with three alternating povidone–iodine and 70% isopropyl alcohol wipes, and the skin was aseptically elevated using blunt forceps, followed by injection of 10 µL of h-UCMSC-Exo using Hamilton needle (MA, 02038, USA, model 1701 RN, 33-gauge neuro syringe, point style 4) subcutaneously. Thus, in this process, the drug accumulated in subcutaneous tissue surrounding the testis.

### 4.8. Serum Hormone Measurements

Testosterone (T) level is an important marker of Leydig cell function; any damage or recovery will be reflected in both serum and testis. The whole blood was collected from mice through cardiac puncture under anesthesia with isoflurane. Serum was separated by centrifuging at 2000× *g* for 15 min at 4 °C and stored at −20 °C until use. The serum hormone levels were measured using a commercially available ELISA kit Cat#EKU05693-96T (Biomatik, DW, USA) for Luteinizing Hormone (LH), cat#EKU04248-96T (Biomatik, USA) for Follicle-Stimulating Hormone (FSH), and cat#EKC40192-96T (Biomatik) for T levels as per the manufacturer’s instructions.

### 4.9. Breeding Experiments

One week after exosome treatment, 4 mice were randomly selected per group for the breeding experiment. Two female mice were bred with one male breeder mouse (C57BL/6). The male and female mice were housed together for 10 days for mating. The presence of a sperm plug in the vagina was checked for mating monitoring. We confirmed that most of the female mice showed a sperm plug within 3 days. After delivery, we counted the average number of pups from each female mouse and compared them among treatment groups. At the end of the experiment, we recorded all the delivered pups per group, their body weight, and any morphological anomalies.

### 4.10. Histological Assays

We harvested testes from mice, fixed in 4% paraformaldehyde, and embedded in paraffin to make formalin-fixed paraffin-embedded tissue (FFPE tissue). FFPE tissue sections were stained with hematoxylin–eosin (H&E) for microscopy. The histology core of the University of Chicago (Chicago, IL, USA) performed sample processing and staining. Terminal deoxynucleotidyl transferase dUTP nick-end labeling (TUNEL) staining assay was performed to detect apoptotic cells in the mouse testis using TUNEL assay (Abcam) on tissue sections (n = 4 per group) according to the manufacturer’s protocol. All images were captured using a Cri Pannoramic SCAN 40X whole slide scanner (3D HISTECH), and the number of TUNEL-positive cells were counted to quantify the apoptotic index using Asperio Image Scope (Leica Biosystem, Wetzlar, Germany).

### 4.11. Statistical Analysis

A two-way ANOVA or nonparametric T test (Mann-Whitney test) with a post-hoc test using GraphPad Prism 9 (GraphPad Software, San Diego, CA, USA) was performed for statical comparisons between groups. All data are presented as the mean ± standard deviation (SD). Statistically significant differences between groups are marked with * (*p* < 0.05), ** (*p* < 0.005), or *** (*p* < 0.0005).

## 5. Conclusions

Exosomes derived from MSCs are emerging as a promising cell-free therapeutic option in the field of regenerative medicine. Our in vitro findings suggest that h-UCMSC-Exo could counteract reproductive toxicity, and the prepubertal mouse study indicates that intra-testicular injections of multiple doses of h-UCMSC-Exo could protect the SSC and niche during chemotherapy, hence preserving fertility. This is relevant, especially for children receiving gonadotoxic drugs. Given the pressing need for effective fertility preservation strategies in young cancer survivors, our preclinical findings are a call to action for pediatric oncologists to explore exosomes as an innovative, cell-free option to protect the germ cell niche before initiating chemotherapy, enhancing future fertility prospects.

## Data Availability

All data are present in the paper and/or the supplementary materials.

## Supplementary Materials

The following supporting information can be downloaded at: https://www.mdpi.com/article/10.3390/ijms25010060/s1.
