# Supplementary material for: Fertility Protection, A Novel Concept: Umbilical Cord Mesenchymal Stem Cell-Derived Exosomes Protect against Chemotherapy-Induced Testicular Cytotoxicity
Source: Int J Mol Sci. 2023 Dec 20;25(1):60. doi: 10.3390/ijms25010060 (PMC10779299; doi:10.3390/ijms25010060)
Supplement: Supplementary file 1 [file ijms-25-00060-s001.zip › ijms-2773889-supplementary.pdf]

a. *In vitro* testicular toxicity model using human Sertoli cells and results

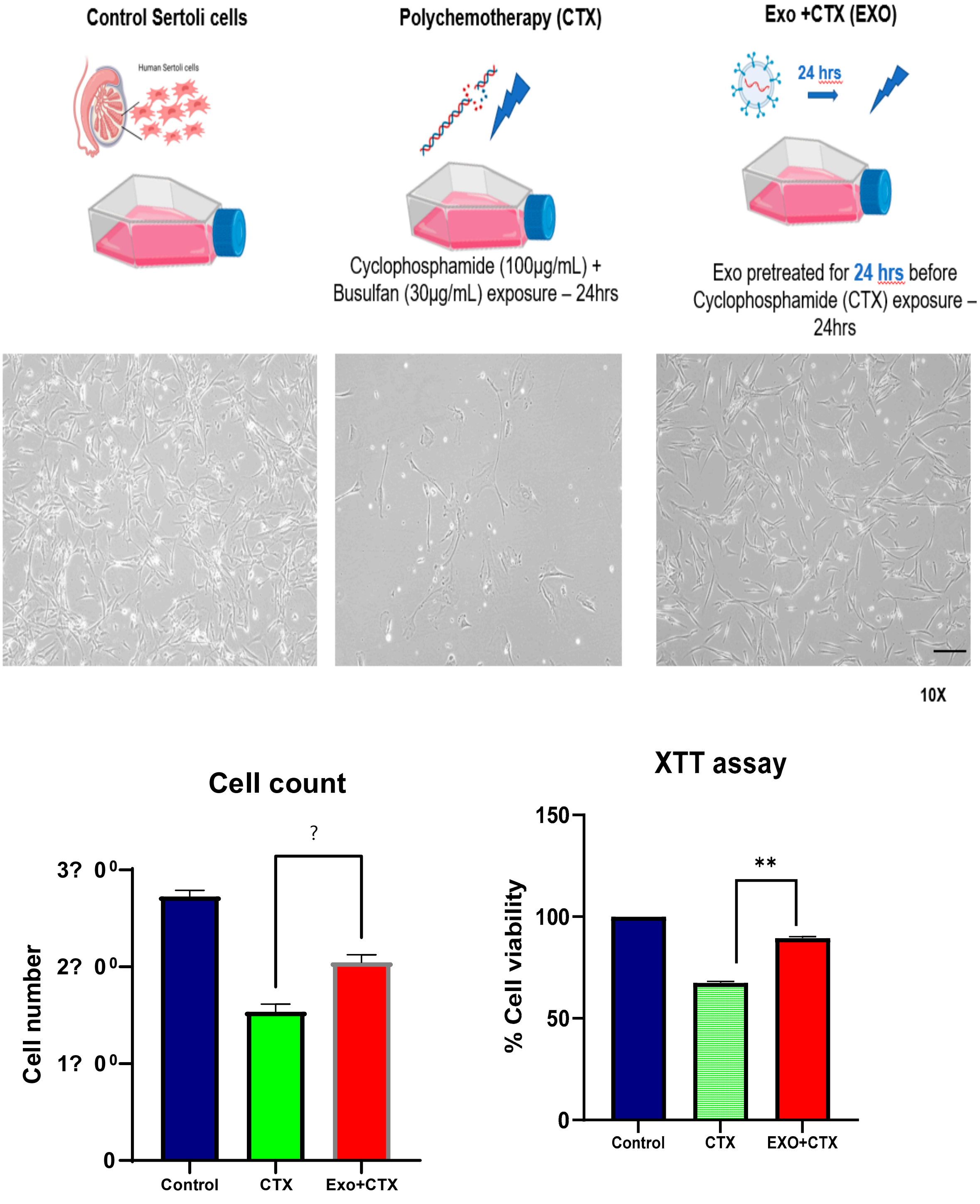

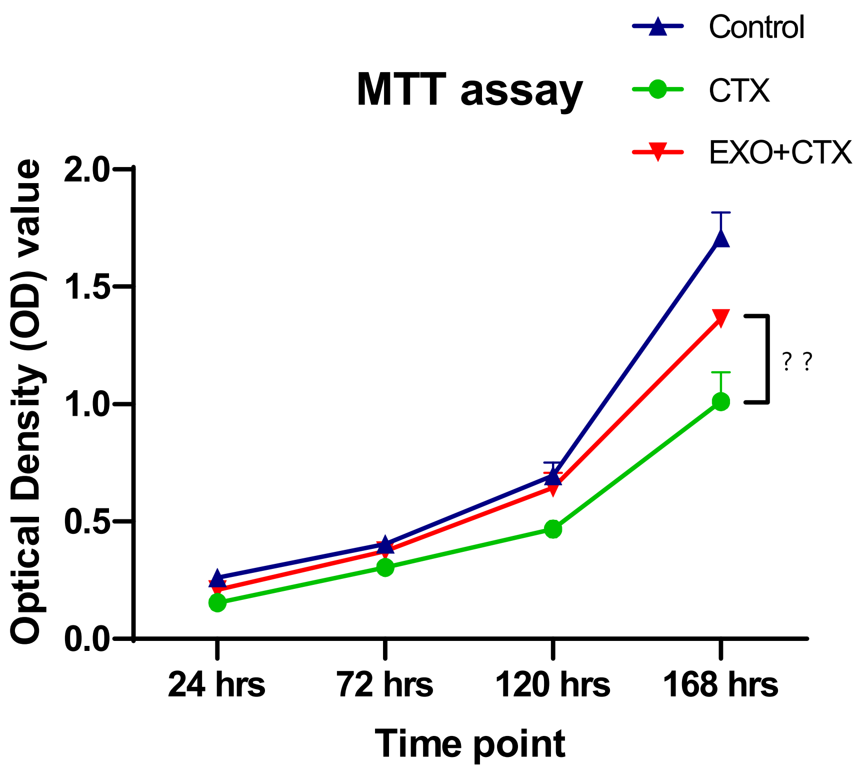

b. Relative gene expression in exosome pre-treated hSer compared to CTX

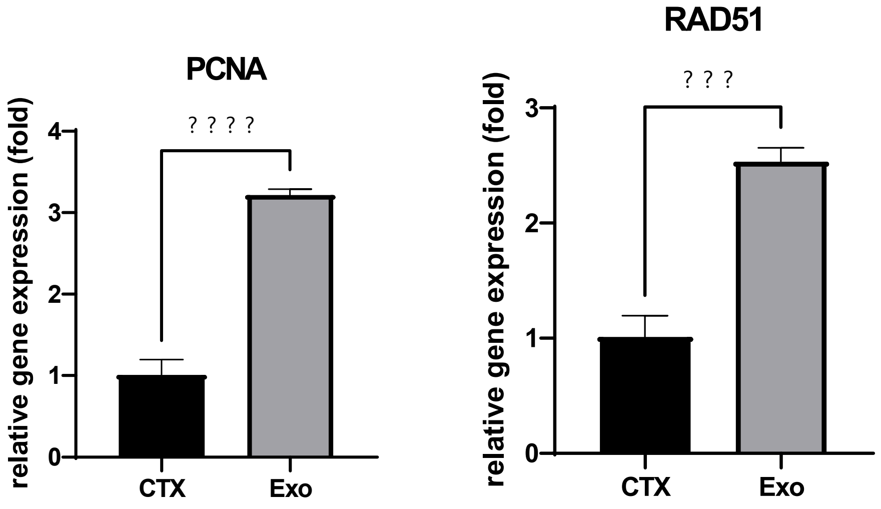

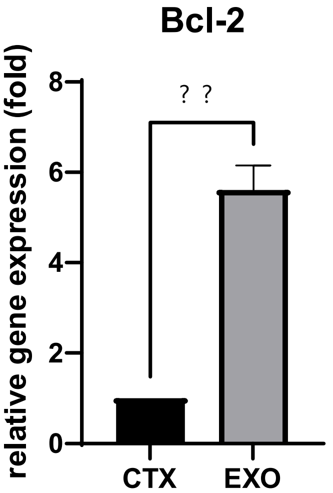
Bcl-2
relative gene expression (fold)
0
2
4
6
8
? ?
CTX
EXO

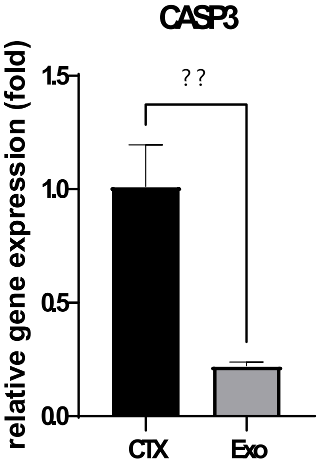
CASP3
relative gene expression (fold)
0.0
0.5
1.0
1.5
??
CTX
Exo

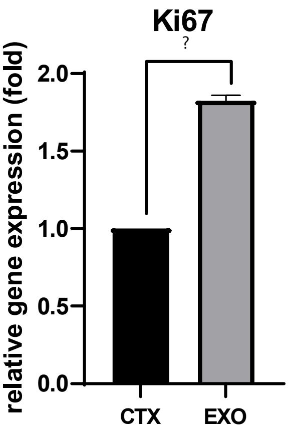
Ki67
relative gene expression (fold)
0.0
0.5
1.0
1.5
2.0
?
CTX
EXO

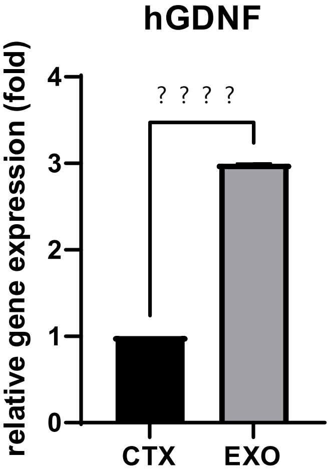
hGDNF
relative gene expression (fold)
0
1
2
3
4
? ? ? ?
CTX
EXO

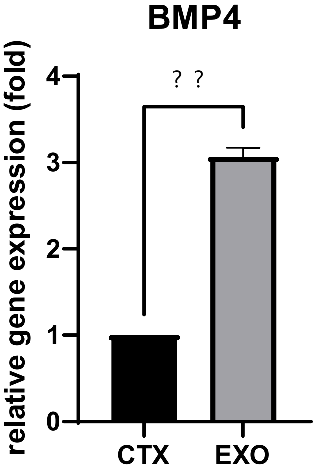
BMP4
relative gene expression (fold)
0
1
2
3
4
? ?
CTX
EXO

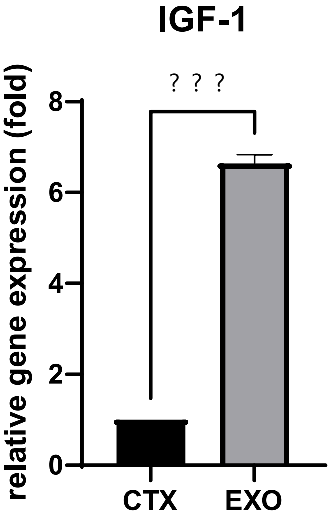
IGF-1
relative gene expression (fold)
0
2
4
6
8
? ? ?
CTX
EXO

c. Gene enrichment pathway

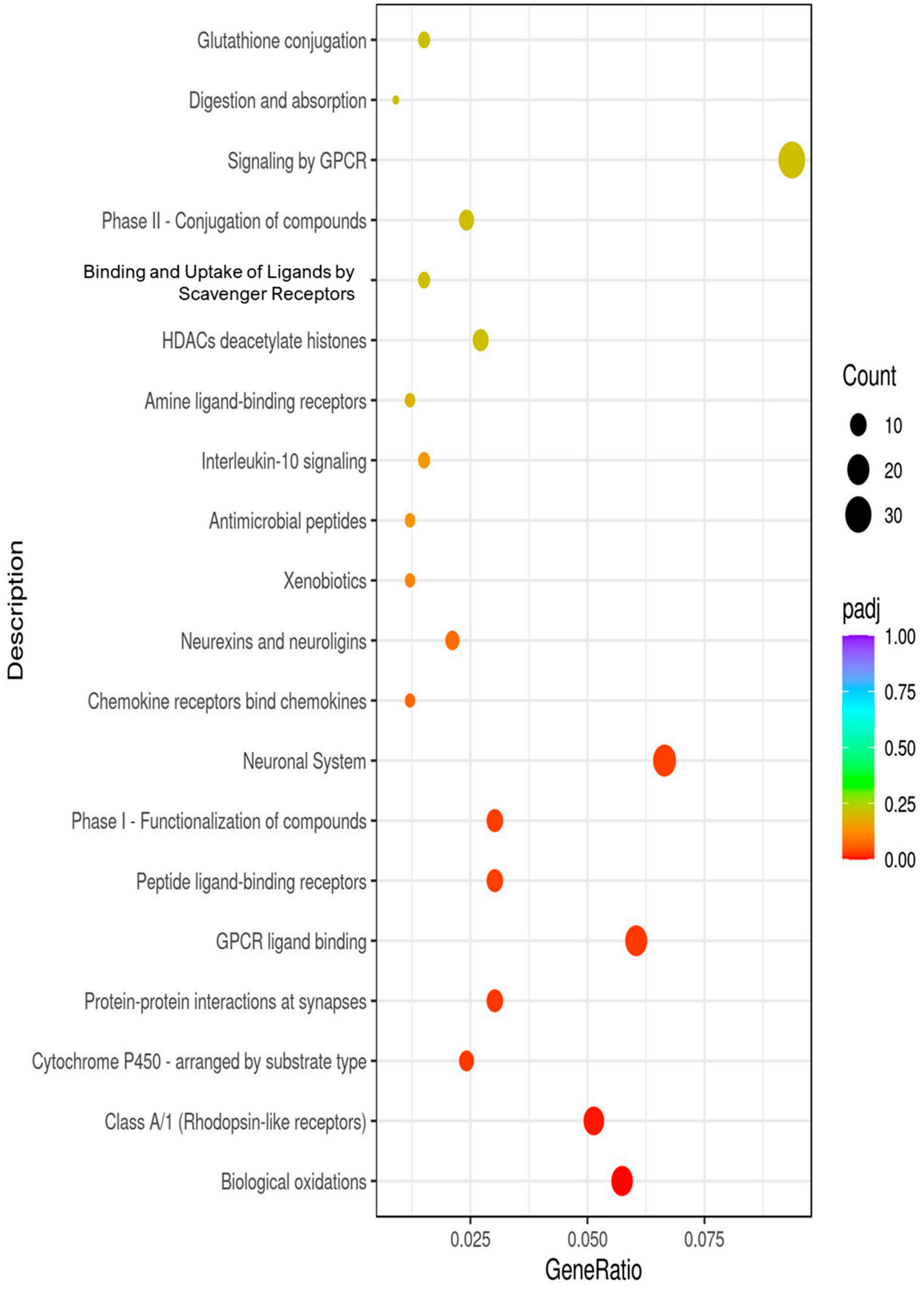

d. Heat map showing differentially expressed genes in control, CTX, and exosome pretreated groups

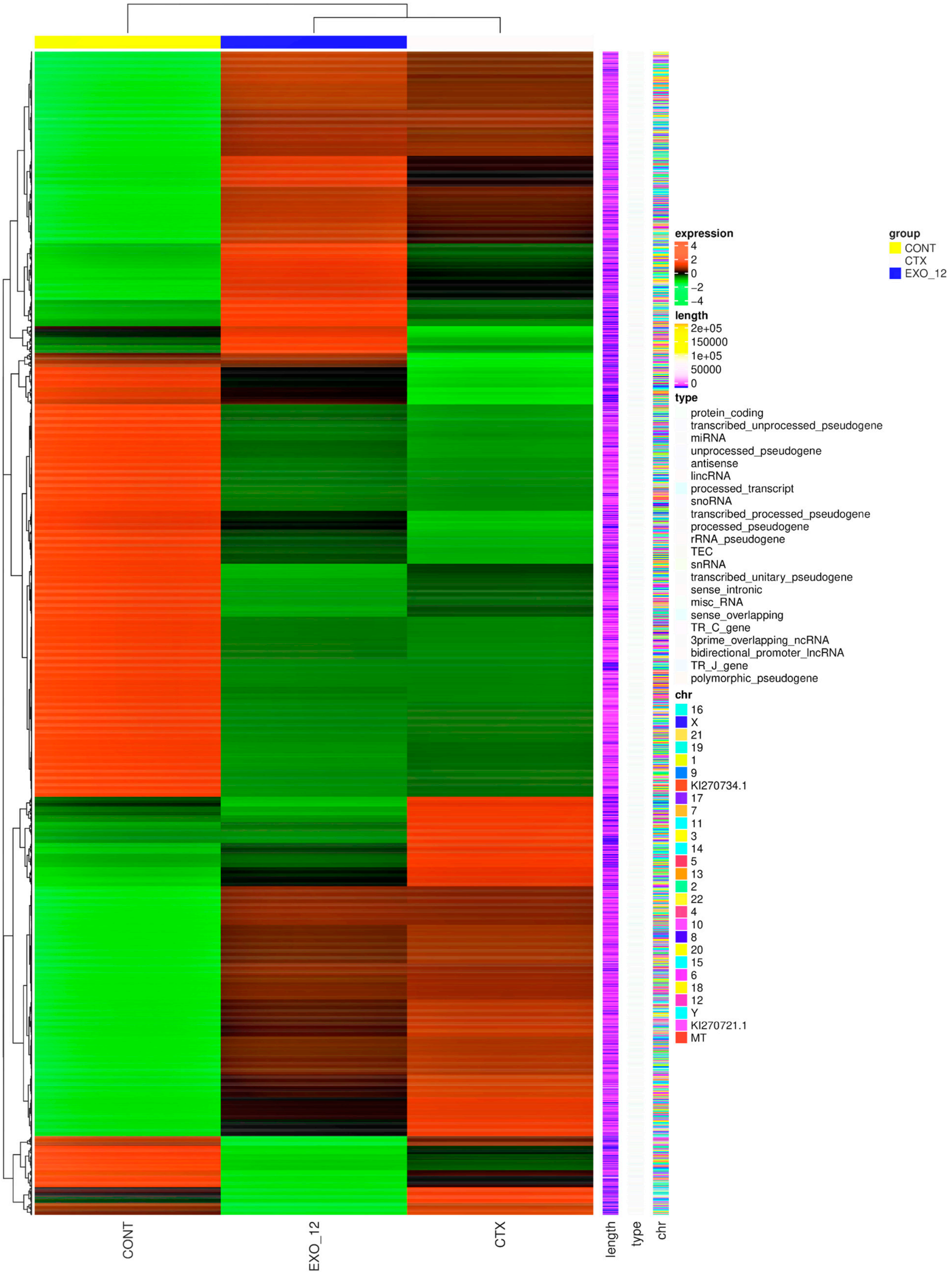

e. The Nano Tracking Particle Analysis (NTA) of exosomes derived from human Umbilical Cord Mesenchymal Stem Cells

NANOSIGHT Naive batch052023 2023-05-22 13-16-23

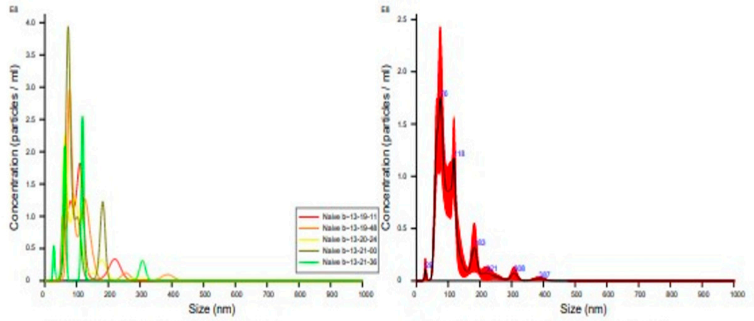

FTLA Concentration / Size graph for Experiment:
Naive batch052023 2023-05-22 13-16-23

Averaged FTLA Concentration / Size for Experiment:
Naive batch052023 2023-05-22 13-16-23
Error bars indicate + / -1 standard error of the mean

Included Files

Naive batch052023 2023-05-22 13-19-11
Naive batch052023 2023-05-22 13-19-48
Naive batch052023 2023-05-22 13-20-24
Naive batch052023 2023-05-22 13-21-00
Naive batch052023 2023-05-22 13-21-36

Details

NTA Version:	NTA 3.4 Build 3.4.4
Script Used:	SOP Standard Measurement 01-16-02PM 22~
Time Captured:	13:16:23 22/05/2023
Operator:	Hans
Pre-treatment:	
Sample Name:	Naive_batch052023
Diluent:	1000
Remarks:	

Capture Settings

Camera Type:	sCMOS
Laser Type:	Green
Camera Level:	13
Slider Shutter:	1232
Slider Gain:	175
FPS	25.0
Number of Frames:	749
Temperature:	26.1 - 26.1 °C
Viscosity:	1.0 cP
Dilution factor:	1 x 10e3
Syringe Pump Speed:	100

Analysis Settings

Detect Threshold:	5
Blur Size:	Auto
Max Jump Distance:	Auto: 15.1 - 27.7 pix

Results

Stats: Merged Data

Mean:	109.6 nm
Mode:	75.2 nm
SD:	56.8 nm
D10:	63.3 nm
D50:	93.5 nm
D90:	182.4 nm

Stats: Mean +/- Standard Error

Mean:	109.9 +/- 3.3 nm
Mode:	88.8 +/- 10.9 nm
SD:	57.3 +/- 6.5 nm
D10:	63.7 +/- 3.0 nm
D50:	97.6 +/- 5.5 nm
D90:	204.4 +/- 25.3 nm
Concentration (Upgrade):	1.04e+10 +/- 1.18e+09 particles/ml
	1.8 +/- 0.2 particles/frame
	2.1 +/- 0.2 centres/frame

Concentration measurements may be unreliable
See summary file for more info

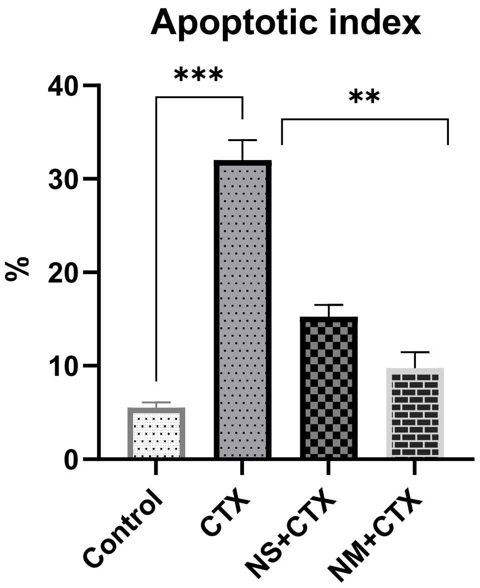

The Johnsen criteria are into a ten-point scoring system for quantifying spermatogenesis according to the profile of the cells encountered along the seminiferous tubules. A Johnsen score of 10 indicates maximum spermatogenesis activity, whereas a score of 1 indicates a complete absence of germ cells.

(*Human Reproduction*, Volume 22, Issue 1, 1 January 2007, Pages 2–16)

Score	Characteristics
10	Complete spermatogenesis with many spermatozoa
9	Many spermatozoa present but germinal epithelium disorganized with marked sloughing or obliteration
8	Only few spermatozoa (<5-10) present in the section
7	No spermatozoa and only many spermatids present
6	No spermatozoa and only few spermatids (<5-10) present
5	No spermatozoa and no spermatids but several or many spermatocytes present
4	Only few spermatocytes (<5) and no spermatids or spermatozoa present
3	Spermatogonia are the only germ cells present
2	No germ cells but Sertoli cells are present
1	No cells in tubular section

Human primers

Sl.no	Primer	sequence(5'-3')	Size(bp)
1	PCNA		
	Forward	GCGTGAACCTCACCAGTATGT	21
	Reverse	TCTTCGGCCCTTAGTGTAATGAT	23
2	Ki67		
	Forward	AGAAGAAGTGGTGCTTCGGAA	21
	Reverse	AGTTTGCGTGGCCTGTACTAA	21
3	MCM2		
	Forward	AGAATCTATGGCGACAG	17
	Reverse	ACCTGC TCTGCCACTAACTG	20
4	CAT		
	Forward	TCCGGGATCTTTTTAACGCCATTG	24
	Reverse	TCGAGCACGGTAGGGACAGTTCAC	24
5	SOD1		
	Forward	GTGGGGAAGCATTAAAGGACTGAC	24
	Reverse	CAATTACACCACAAGCCAAACGAC	24
6	SOD2		
	Forward	CTCCCCGACCTGCCCTACGACTAC	24
	Reverse	AAACCAAGCCAACCCCAACCTGAG	24
7	GPX1		
	Forward	GCGGCGGCCCAGTCGGTGTA	20
	Reverse	GAGCTTGGGGTCGGTCATAA	20
8	GDNF		
	Forward	GGCAGTGCTTCCTAGAAGAGA	21
	Reverse	AAGACACAACCCCGGTTTTTG	21
9	SCF		
	Forward	AATCCTCTCGTCAAAACTGAAGG	23
	Reverse	CCATCTCGCTTATCCAACAATGA	23
10	BMP4		
	Forward	ATGATTCCTGGTAACCGAATGC	22
	Reverse	CCCCGTCTCAGGTATCAAACT	21
11	WT1		
	Forward	CACAGCACAGGGTACGAGAG	20
	Reverse	CAAGAGTCGGGGCTACTCCA	20
12	GATA4		
	Forward	CGACACCCCAATCTCGATATG	21
	Reverse	GTTGCACAGATAGTGACCCGT	21
13	SOX9		
	Forward	AGCGAACGCACATCAAGAC	19
	Reverse	CTGTAGGCGATCTGTTGGGG	20
14	ZO1		

	Forward	ACCAGTAAGTCGTCCTGATCC	21
	Reverse	TCGGCCAAATCTTCTCACTCC	21
15	OCLN		
	Forward	ACAAGCGGTTTTATCCAGAGTC	22
	Reverse	GTCATCCACAGGCGAAGTTAAT	22
16	EGF		
	Forward	TCCTCACCCGATAATGGTGGA	21
	Reverse	CCAGGAAAGCAATCACATTCCC	22
17	LIF		
	Forward	CCAACGTGACGGACTTCCC	19
	Reverse	TACACGACTATGCGGTACAGC	21
18	IGF1		
	Forward	GCTCTTCAGTTCGTGTGTGGA	21
	Reverse	GCCTCCTTAGATCACAGCTCC	21
19	INHBB		
	Forward	CGGGTCCGCCTATACTTCTTC	21
	Reverse	CGTAGGGCAGGAGTTTCAGG	20
20	CXCL12		
	Forward	ATTCTCAACACTCCAAACTGTGC	23
	Reverse	ACTTTAGCTTCGGGTCAATGC	21
21	ETV5		
	Forward	TCAGCAAGTCCCTTTTATGGTC	22
	Reverse	GCTCTTCAGAATCGTGAGCCA	21
22	FSHR		
	Forward	TCTGTCACTGCTCTAACAGGG	21
	Reverse	TGCACCTTTTTGGATGACTCG	21
23	Vimentin		
	Forward	AGTCCACTGAGTACCGGAGAC	21
	Reverse	CATTTCACGCATCTGGCGTTC	21
24	AR		
	Forward	GACGACCAGATGGCTGTCATT	21
	Reverse	GGGCGAAGTAGAGCATCCT	19
25	GJA1		
	Forward	TGGTAAGGTGAAAATGCGAGG	21
	Reverse	GCACTCAAGCTGAATCCATAGAT	23
26	CDH2		
	Forward	TGCGGTACAGTGTAACTGGG	20
	Reverse	GAAACCGGGCTATCTGCTCG	20
27	Beta actin		
	Forward	CATGTACGTTGCTATCCAGGC	21
	Reverse	CTCCTTAATGTCACGCACGAT	21
28	LRP6		
	Forward	ACGATTGTAGTTGGAGGCTTG	21

	Reverse	ATGGCTTCTTCGCTGACATCA	21
29	b - *catenin*		
	Forward	CATCTACACAGTTTGATGCTGCT	23
	Reverse	GCAGTTTTGTCAGTTCAGGGA	21
30	c-Myc		
	Forward	GTCAAGAGGCGAACACACAAC	21
	Reverse	TTGGACGGACAGGATGTATGC	21
31	Cyclin D1		
	Forward	GCTGCGAAGTGGAAACCATC	20
	Reverse	CCTCCTTCTGCACACATTTGAA	22

Mouse primers

**Sl.no**	**Gene**	**Accession no**	**Primer Sequence**
1	Pou5f1	NM_013633	Forward 5'-cgggctgggtggatcctcga-3'<br>Reverse 5'-ttcacggcattggggcggtc-3'
2	Zbtb 16 (Plzf)	NM_001033324	Forward 5'-gcaggagccagcaaaggcga-3'<br>Reverse 5'-gcagagaccccagggagggg-3'
3	Gfra-1	NM_010279	Forward 5'-ggctaggaggaggagatgct-3'<br>Reverse 5'-ctggatgtgaccagggactt-3'
4	Bcl6b	NM_007528	Forward 5'-gcagcagtgaagaaggaacc-3'<br>Reverse 5'-agccacagcctcacagttct-3'
5	Etv5	NM_023794	Forward 5'-gggagagacaaaaaccacca-3'<br>Reverse 5'-atgggtgtgcagtttcttcc-3'
6	Stra8	NM_009292	Forward 5'- ttcctgcgtgttccacaagt- 3'<br>Reverse 5'- tacctgccactttgaggctg- 3'
7	Crem	NM_001110854	Forward 5'-tgaaactgatgaggagactgacc-3'<br>Reverse 5'- attttcaagcacagccacacg- 3'
8	Mea 1	NM_010787	Forward 5'-gaggaaataccaacgccgaga-3'<br>Reverse 5'-ctgtaggaccctgatgtggc-3'
9	Sycp1	NM_011516	Forward 5'-catgctcgaacaggttgcta-3'<br>Reverse 5'-ttcgctgggcttcaattatc-3'
10	Bak 1	NM_007523	Forward 5'-ggtgacctgctttttggctg-3'<br>Reverse 5'-taccacgaattggcccaaca-3'
11	Bad	NM_007522	Forward 5'-agcgtacgcacacctatcct-3'

			Reverse 5’-caatggtcgttgcgatggtt-3’
12	Bcl2	NM_177410	Forward 5’-gcgtcaacagggagatgtca-3’<br>Reverse 5’- ccagaatccactcacacccc-3’
13	Mcl1	NM_008562	Forward 5’- tgccagcttgggagtgattt<br>Reverse 5’- actgcgctctccaagtcttc-3’
14	Cdk10	NM_194446	Forward 5’-gaaggtttcttcaccgtgcc-3’<br>Reverse 5’-ccacaatgtttggatggcgg-3’
15	Cul3	NM_016716	Forward 5’-cctggcaagaagactgctca-3’<br>Reverse 5’-ccccgttgcctgtagatgtt-3’
16	Plk1	NM_011121	Forward 5’-tggagcaacttcggcatcat-3’<br>Reverse 5’-gcctgcgaacacctcttttg- 3’
17	CD2		Forward 5'-gccacctggatgctagagg-3'<br>Reverse 5'- ggcttcacagagttgtcgg-3'
